# Avelumab-Induced Scleroderma in a Patient with Metastatic Squamous Cell Carcinoma of the Lung

**DOI:** 10.1155/2022/5360113

**Published:** 2022-12-15

**Authors:** Jeffrey L. Dobrzynski, Blake E. Vest, Brian L. Swick

**Affiliations:** ^1^University of Iowa Roy J. and Lucille A. Carver College of Medicine, 375 Newton Rd, Iowa City, IA 52242, USA; ^2^University of Iowa Hospitals and Clinics, Dermatology, 200 Hawkins Drive, Iowa City, IA 52242, USA

## Abstract

Immune checkpoint inhibitors are associated with a spectrum of cutaneous immune-related adverse events. While maculopapular eruptions are the most common cutaneous adverse event, scleroderma can rarely develop. Herein, we report a case of new-onset scleroderma associated with avelumab treatment in the setting of metastatic squamous cell carcinoma of the lung. The pathophysiology of immune checkpoint inhibitor-induced scleroderma is not completely understood. A proposed mechanism is discussed along with the clinical presentation of symptoms and associated therapeutic response in cancer treatment. This case contributes to the few existing reports of immune checkpoint inhibitor-induced scleroderma to better understand the implications in the management of cutaneous immune-related adverse events.

## 1. Introduction

Immune checkpoint inhibitors (ICIs) are immunotherapy agents effective in treating a variety of cancers [1]. ICIs utilize antibodies that target immune checkpoint proteins such as the CTLA-4 receptor, PD-1 receptor, or PD-1 ligand. These checkpoint proteins function to downregulate the immune system. By blocking these targets, ICIs act to increase immune system activity and increase antitumor immunity. Despite their efficacy in treating advanced cancers, ICIs are associated with a spectrum of immune-related adverse events (irAEs) [1–3]. Of these, cutaneous immune-related adverse events (cirAEs) are the most common, affecting 20% to 40% of all patients treated with ICIs [4]. Although maculopapular eruptions occur most frequently, rarely scleroderma can develop [3]. Here we report a case of scleroderma induced by the PD-L1 inhibitor, avelumab.

## 2. Case Report

**Figure 1:**
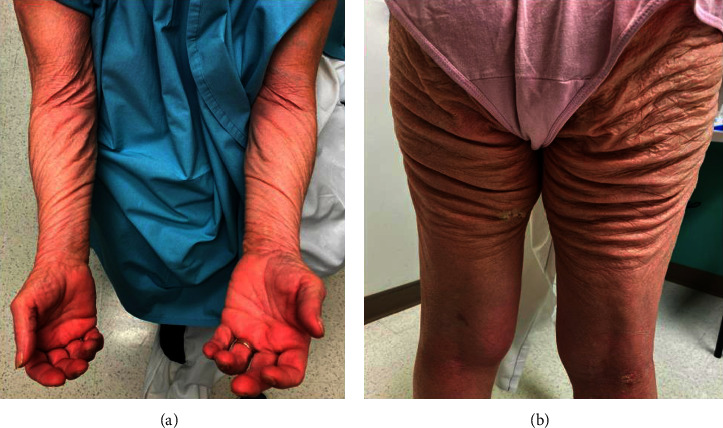
Clinical photographs show areas of diffuse indurated, sagging skin, with decreased elasticity and fine scaling. (a) Ventral forearms and wrists. (b) Posterior thighs.

A 67-year-old female with metastatic squamous cell carcinoma of the lung on avelumab and utomilumab for almost 3 years presented with a 2-month history of a worsening rash. The rash started as rough spots on the volar wrists and posterior knees that progressed into diffuse areas of loose, sagging skin involving the trunk, extremities, and intertriginous areas (Figure 1). Immunotherapy was withheld due to the development of skin lesions.

**Figure 2:**
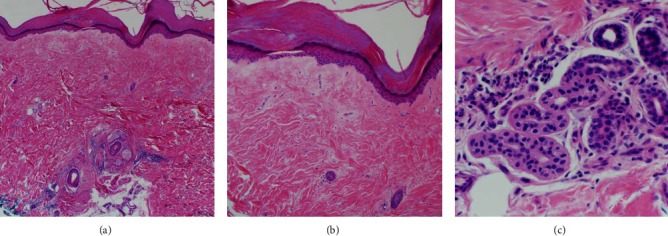
Punch biopsy showed thickened dermal collagen bundles, eccrine gland entrapment, and sparse plasma cells in the deep reticular dermis. (hematoxylin-eosin stain, original magnifications (a) ×40, (b) ×100, and (c) ×200).

Punch biopsies of the calf and thigh both showed thickened dermal collagen bundles, eccrine gland entrapment, and sparse, plasma cell-rich, chronic inflammation along the dermal-subcutaneous junction (Figure 2). Elastichrome stain exhibited normal elastin fibers. Serologic testing revealed elevated ANA of 1:320 with a coarse speckled pattern, but negative Scl-70, centromere, SM/RNP, RNA polymerase III, and PM-Scl-100 antibodies. These findings were consistent with a diagnosis of avelumab-induced scleroderma.

The patient was treated with methotrexate 17.5 mg weekly and a prednisone taper, starting at 60 mg daily and decreasing by 5 mg every week. The patient had neither improvement nor worsening on a one-month follow-up. Two months after discontinuation of immunotherapy, a new lesion of the left upper lobe of the lung was identified on chest CT, and the biopsy demonstrated small cell carcinoma. A new primary lesion was favored over the transformation of her original malignancy.

## 3. Discussion

Interaction between PD-L1 and the PD-1 receptor plays a key role in dermal thickening and the development of skin sclerosis and fibrosis in patients with scleroderma [5]. Although the pathophysiology is not completely understood, it is proposed that PD-1/PD-L1 inhibitor-induced scleroderma may result due to the development of Th1, Th2, and Th17 cells that induce profibrotic type 2 macrophages causing fibroblast activation and fibrosis [5].

A recent review of ICI-induced scleroderma found only 35 reported cases of ICI-induced scleroderma, with 30 cases caused by the PD-1 inhibitors pembrolizumab or nivolumab, three caused by the CTLA-4 inhibitor ipilimumab, and 2 caused by the PD-L1 inhibitors atezolizumab and durvalumab [5]. ICI-induced scleroderma may result in atypical presentations depending on the specific medication being used. Nivolumab seems to cause localized symptoms compared to pembrolizumab, which more often causes generalized skin thickening [5]. As opposed to the shiny, taught skin that spreads distally from the trunk seen in typical systemic scleroderma, our patient developed more distal extremity followed by truncal skin thickening with a wrinkled appearance, further demonstrating the variability in the clinical presentation of scleroderma induced by different ICIs. Additionally, drug-induced scleroderma lacks Raynaud's phenomenon and the internal organ involvement seen in systemic sclerosis [6].

Utomilumab is a fully human immunoglobulin G2 monoclonal antibody agonist of the T-cell costimulatory receptor 4-1BB/CD137. This agent is under investigation for the treatment of solid organ and hematologic malignancies. Overall, it has a favorable safety profile with no dose-limiting toxicities reported in a phase Ib trial [7]. Although less likely, the contribution of utomilumab to this patient's cutaneous adverse event cannot be completely excluded. Applying the WHO-UMC causality criteria, there is a probable/likely degree of association between avelumab and scleroderma observed in this case [8].

Multiple studies have shown that the development of irAEs is associated with increased therapeutic response in cancer treatment [9]. Two months after discontinuation of avelumab our patient had stable to improved cancer burden; however, she developed a new primary tumor diagnosed as small cell carcinoma of the lung. This was treated with stereotactic body radiation therapy and a follow-up CT of the chest, abdomen, and pelvis showed no residual or new tumors in the lungs. The patient's scleroderma was initially stable after the discontinuation of immunotherapy and initiation of methotrexate and prednisone. However, she developed a progression of scleroderma on the bilateral ankles after tapering off prednisone. These symptoms stabilized after restarting prednisone. Although high-dose systemic corticosteroids are often avoided when treating systemic sclerosis due to the risk of renal crisis, this along with methotrexate are mainstay treatments for the isolated skin findings without internal organ involvement seen in drug-induced scleroderma [6]. This case of avelumab-induced scleroderma contributes to the few existing reports of ICI-induced scleroderma to better understand the implications in the management of both cirAEs and the underlying malignancy.
